# Characterizing the double‐sided cascade of care for adolescents living with HIV transitioning to adulthood across Southern Africa

**DOI:** 10.1002/jia2.25447

**Published:** 2020-01-30

**Authors:** Priscilla R Tsondai, Annette H Sohn, Sam Phiri, Kombatende Sikombe, Shobna Sawry, Cleophas Chimbetete, Geoffrey Fatti, Michael A Hobbins, Karl‐Günter Technau, Helena Rabie, Jonathan Bernheimer, Matthew P Fox, Ali Judd, Intira J Collins, Mary‐Ann Davies, for the International epidemiology to Evaluate AIDS Southern Africa (IeDEA‐SA) Collaboration, Christopher Hoffmann, Michael Vinikoor, Robin Wood, Andrew Boulle, Geoffrey Fatti, Sam Phiri, Janet Giddy, Cleophas Chimbetete, Brian Eley, Olatunbosun Faturiyele, Michael Hobbins, Matthew Fox, Hans Prozesky, Karl Technau, Shobna Sawry

**Affiliations:** ^1^ Centre for Infectious Disease Epidemiology and Research School of Public Health and Family Medicine Faculty of Health Sciences University of Cape Town Cape Town South Africa; ^2^ TREAT Asia/amfAR – The Foundation for AIDS Research Bangkok Thailand; ^3^ Lighthouse Trust Clinic Lilongwe Malawi; ^4^ Centre for Infectious Disease Research in Zambia Lusaka Zambia; ^5^ Harriet Shezi Children's Clinic Wits Reproductive Health and HIV Research Unit University of Witwatersrand Johannesburg South Africa; ^6^ Newlands Clinic Harare Zimbabwe; ^7^ Kheth'Impilo Cape Town South Africa; ^8^ Division of Epidemiology and Biostatistics Department of Global Health Faculty of Medicine and Health Sciences Stellenbosch University Cape Town South Africa; ^9^ SolidarMed Luzern Switzerland; ^10^ Empilweni Services and Research Unit Department of Paediatrics & Child Health Rahima Moosa Mother and Child Hospital Faculty of Health Sciences University of the Witwatersrand Johannesburg South Africa; ^11^ Department of Paediatrics and Child Health Tygerberg Academic Hospital University of Stellenbosch Stellenbosch South Africa; ^12^ Médecins Sans Frontiers Khayelitsha South Africa; ^13^ Department of Global Health and Department of Epidemiology Boston University School of Public Health Boston MA USA; ^14^ Health Economics and Epidemiology Research Office Faculty of Health Sciences University of Witwatersrand Johannesburg South Africa; ^15^ MRC Clinical Trials Unit at UCL University College London (UCL) London United Kingdom

**Keywords:** HIV, adolescents, youth, healthcare transition, retention, viral suppression, cascade of care

## Abstract

**Introduction:**

As adolescents and young people living with HIV (AYLH) age, they face a “transition cascade,” a series of steps associated with transitions in their care as they become responsible for their own healthcare. In high‐income countries, this usually includes transfer from predominantly paediatric/adolescent to adult clinics. In sub‐Saharan Africa, paediatric HIV care is mostly provided in decentralized, non‐specialist primary care clinics, where “transition” may not necessarily include transfer of care but entails becoming more autonomous for one's HIV care. Using different age thresholds as proxies for when “transition” to autonomy might occur, we evaluated pre‐ and post‐transition outcomes among AYLH.

**Methods:**

We included AYLH aged <16 years at enrolment, receiving antiretroviral therapy (ART) within International epidemiology Databases to Evaluate AIDS Southern Africa (IeDEA‐SA) sites (2004 to 2017) with no history of transferring care. Using the ages of 16, 18, 20 and 22 years as proxies for “transition to autonomy,” we compared the outcomes: no gap in care (≥2 clinic visits) and viral suppression (HIV‐RNA <400 copies/mL) in the 12 months before and after each age threshold. Using log‐binomial regression, we examined factors associated with no gap in care (retention) in the 12 months post‐transition.

**Results:**

A total of 5516 AYLH from 16 sites were included at “transition” age 16 (transition‐16y), 3864 at 18 (transition‐18y), 1463 at 20 (transition‐20y) and 440 at 22 years (transition‐22y). At transition‐18y, in the 12 months pre‐ and post‐transition, 83% versus 74% of AYLH had no gap in care (difference 9.3 (95% confidence interval (CI) 7.8 to 10.9)); while 65% versus 62% were virally suppressed (difference 2.7 (−1.0 to 6.5%)). The strongest predictor of being retained post‐transition was having no gap in the preceding year, across all transition age thresholds (transition‐16y: adjusted risk ratio (aRR) 1.72; 95% CI (1.60 to 1.86); transition‐18y: aRR 1.76 (1.61 to 1.92); transition‐20y: aRR 1.75 (1.53 to 2.01); transition‐22y: aRR 1.47; (1.21 to 1.78)).

**Conclusions:**

AYLH with gaps in care need targeted support to prevent non‐retention as they take on greater responsibility for their healthcare. Interventions to increase virologic suppression rates are necessary for all AYLH ageing to adulthood.

## 1 Introduction

There is a growing cohort of adolescents and young adults living with HIV (AYLH), largely due to the increasing number of children with perinatally acquired HIV surviving into adolescence and adulthood, combined with a growing number of youth with non‐perinatally acquired HIV. In 2017, nearly three million youth (aged 15 to 24 years) were living with HIV in sub‐Saharan Africa 1. As these young people become adults, they are increasingly expected to become responsible for their own healthcare and progressively required to start setting up their own clinic appointments and be responsible for collecting and taking their antiretroviral therapy (ART).

Clinical outcomes and retention among AYLH have generally been poorer when compared to young children and older adults 2, 3, 4, 5, 6. In settings where paediatric HIV care is provided within specialized paediatric facilities, adolescents and young adults have to be transferred out to adult HIV clinics as they age. Research from North America and Europe has shown that during this process, not all youth transferred to adult clinics successfully continue care 7, 8, 9, 10, 11, 12. There are very limited data on transition outcomes among AYLH in sub‐Saharan Africa 13 and a paucity of evidence as to the outcomes of adolescents as they grow older in settings where transition to adulthood necessitates taking on greater responsibility for one's healthcare but is *not* accompanied by physical transfer of care from a paediatric/adolescent clinic to a distinct adult HIV clinic. This could be the most common scenario within sub‐Saharan Africa as only a third of facilities included in a situational analysis of facilities within sub‐Saharan Africa reported attending to adolescents separately from adult and/or paediatric patients 14. Also, most studies have reported on outcomes *after* transition, with few studies 15, 16 describing engagement in care among transitioning youth in the period *before* transition. Because they often have an extended period of HIV care and ART prior to transition, unlike conventional HIV cascades that start after diagnosis, the adolescent transition cascade needs to be double‐sided, comparing outcomes both before and after transition 17.

We sought to evaluate gaps in care and viral suppression in AYLH in the year before and after their 16^th^, 18^th^, 20^th^ and 22^nd^ birthdays, using these different age thresholds as proxies for when “transition” to autonomy may occur in the context where adolescents remain at the same facility through to adulthood.

## 2 Methods

### 2.1 Study population

We analysed prospectively collected data of AYLH who were receiving ART within International epidemiology Databases to Evaluate AIDS Southern Africa (IeDEA‐SA) sites between 2004 and 2017. The IeDEA‐SA cohort is an NIH‐funded collaboration which collects de‐identified routine patient data on demographics, antiretroviral drugs, clinical contacts and laboratory tests from cohorts within six Southern African countries: Lesotho, Malawi, Mozambique, South Africa, Zambia and Zimbabwe 18. These data are transferred annually to the IeDEA‐SA Data Centers at the Universities of Cape Town, South Africa, and Bern, Switzerland, for inclusion in combined analyses using a standard data transfer format.

### 2.2 Ethics

All cohorts contributing data to IeDEA‐SA have ethics approval to contribute de‐identified data to the IeDEA‐SA Data Centers. Their respective institutional review boards have granted waivers of informed consent as the analyses use data collected as part of routine patient care. The IeDEA‐SA Data Centers have ethics approval to combine and conduct analyses on the de‐identified data.

### 2.3 Outcomes and analysis

**Figure 1:**
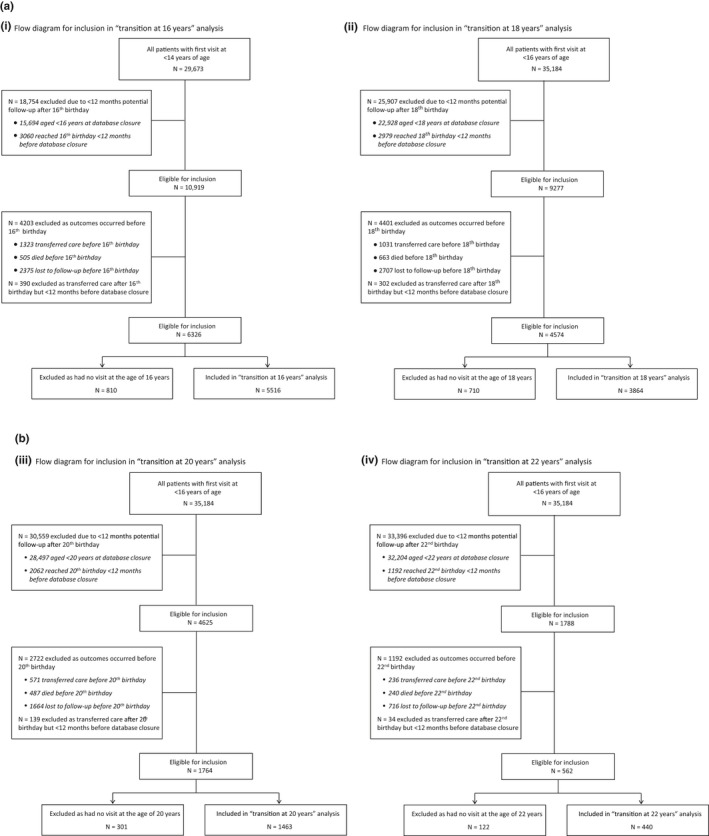
(a) Flow diagram for inclusion in: (i) “transition at 16 years” and (ii) “transition at 18 years” analyses. (b) Flow diagram for inclusion in: (iii) “transition at 20 years” and (iv) “transition at 22 years” analyses.

We assessed “transition” at the ages of 16, 18, 20 and 22 years, with a special focus on the age of 18 years, as this is the legal age of adulthood in most Southern African countries. For this analysis, age was used as a proxy for “transition” to adulthood because in the majority of facilities included, growing up into adulthood is not usually accompanied by physical transfer of care to a distinct adult HIV clinic. To be included in the analysis of “transition” at age 18 years (transition‐18y), patients had to have been enrolled into HIV care before the age of 16 years, as patients enrolling at older ages (e.g. at the age of 20 years) would be responsible for their own healthcare at enrolment in routine care settings; have at least one visit at the age of 18 years within a window of six months before and after their 18^th^ birthday (i.e. have at least one visit between the ages of 17.5 to 18.4 years); been on ART by their 18^th^ birthday; and not transferred to a different facility as they aged up. In addition, the date of a patient's 18^th^ birthday had to be at least one year before the closure of their cohort database to allow for evaluation of retention at one‐year post‐transition (Figure 1a). These criteria were adjusted accordingly for the analyses at 16 years (transition‐16y), at 20 years (transition‐20y) and at 22 years (transition‐22y) (Figures 1a and 1b). While the transition‐18y, transition‐20y and transition‐22y analyses included patients enrolled into HIV care before the age of 16 years, the transition‐16y analysis only included patients enrolled into care before the age of 14 years so that engagement in the year prior to transition could be assessed. When assessing viral suppression outcomes, we only included patients in care within facilities with routine annual viral load testing.

The primary objectives were to compare the proportion of patients (a) with no gap in care in the 12 months before and 12 months after the respective age threshold, and (b) virally suppressed in the 18 months before and 18 months after each transition‐age threshold. No gap in care was defined as having at least two clinic visits more than two months apart in the ± 12‐month period. Viral suppression was defined as an HIV‐RNA viral load < 400 copies/mL in the ±18‐month period. We used an 18‐month window to assess viral suppression as routine viral load testing is recommended annually and we wanted to allow enough time on either side of the transition age for a viral load to be done and documented. Secondary objectives were to assess changes in the proportion of patients with no gap in care in the 12‐month period post‐transition as the age of enrolment into HIV care increased (<10, 10 to 14, and 15 to 16 years), and to evaluate predictors of having no gap in care in the post‐transition period. Adolescents with no gap in care after the respective transition‐age threshold were considered “retained.” As our primary analysis may have favoured the pre‐transition period since adolescents had to have a visit within a year of the relevant age threshold to be included, sensitivity analyses examining these outcomes among only AYLH retained at the time of database closure were conducted. In addition, an analysis assuming “transition” at the age of 15 years (transition‐15y) was conducted given that the age of 15 years is often used as the cut‐off point by many national governments when reporting HIV data.

Characteristics of AYLH for each analysis were described by frequencies for categorical variables and as medians and inter‐quartile ranges for continuous variables. As the mode of infection was not available in our data, we considered adolescents who had enrolled into HIV care before the age of 10 years as likely perinatally infected 19. We investigated whether there were any differences in the proportion of patients with no gap in care and proportion virally suppressed *before* and *after* each respective age threshold which we termed as the pre‐ and post‐transition periods. Predictors of being retained in the 12 months post‐transition were assessed using log‐binomial regression models. In the adjusted models we included the following pre‐determined covariates: sex (male or female), age at first entry into HIV care (<10, 10 to 14, or 15 to 16 years), total number of patients transitioning at the same time in the clinic (≤50, 51 to 100, 101 to 200, or >200) and having a gap in care in the 12‐month period pre‐transition (gap in care vs. no gap in care).

All statistical analyses were conducted in Stata 15.0 (STATA Corporation, College Station, Texas, USA).

## 3 Results

The analyses included 5516 (51% female) AYLH at transition‐16y, 3864 (53% female) at transition‐18y, 1463 (54% female) at transition‐20y and 440 (59% female) at transition‐22y thresholds (Table 1 and Figures 1a and 1b). Across transition‐age cohorts, the majority enrolled into HIV care (61% to 68%) and started ART (50% to 67%) between the ages of 10 and 14 years. The proportion of adolescents assumed to have perinatal HIV (enrolled into HIV care aged <10 years) decreased with increasing transition‐age threshold from 34% (transition‐16y) to 2% (transition‐22y) (Table 1).

**Table 1:** Characteristics of adolescents at different transition^a^ age thresholds

Characteristics	“Transition” at 16 years	“Transition” at 18 years	“Transition” at 20 years	“Transition” at 22 years
Number	5516	3864	1463	440
Female, n (%)	2831 (51)	2044 (53)	796 (54)	258 (59)
Age at enrolment in HIV care (years), n (%)				
<10	1884 (34)	543 (14)	67 (5)	9 (2)
10 to 14	3632 (66)	2489 (64)	993 (68)	268 (61)
15 to 16	─	832 (22)	403 (27)	163 (37)
Median (IQR) age at enrolment in HIV care (years)	11.2 (9.2 to 12.7)	13.3 (11.3 to 14.8)	13.9 (12.4 to 15.1)	14.5 (13.4 to 15.3)
Year of “transition,” n (%)				
<2010	687 (12)	407 (11)	63 (4)	6 (1)
2010 to 2012	2025 (37)	1453 (38)	496 (34)	109 (25)
2013 to 2014	2245 (41)	1599 (41)	757 (52)	278 (63)
2015 to 2016	559 (10)	405 (10)	147 (10)	47 (11)
Year of ART start, n (%)				
≤2004	513 (9)	321 (8)	160 (11)	66 (15)
2005 to 2009	3795 (70)	2615 (69)	1103 (76)	337 (78)
≥2010	1136 (21)	852 (23)	180 (12)	30 (7)
Age at ART start (years), n (%)				
<10	1615 (30)	444 (12)	54 (4)	4 (1)
10 to 14	3632 (67)	2330 (61)	880 (61)	218 (50)
≥15	197 (4)	1014 (27)	509 (35)	211 (49)
Median (IQR) age at ART start (years)	11.5 (9.6 to 13.1)	13.5 (11.6 to 15.1)	14.2 (12.7 to 15.5)	15.0 (13.8 to 15.8)
Median (IQR) duration on ART at time of “transition” (years)	4.5 (3.0 to 6.5)	4.5 (2.9 to 6.4)	5.8 (4.5 to 7.3)	7.1 (6.2 to 8.2)
Median (IQR) duration on ART at time of “transition” by age at enrolment in HIV care (years)				
<10	7.2 (6.3 to 8.4)	8.7 (8.0 to 9.7)	10.4 (10.0 to 11.5)	11.2 (10.0 to 12.3)
10 to 14	3.5 (2.5 to 4.6)	4.7 (3.7 to 6.0)	6.4 (5.4 to 7.5)	7.8 (7.1 to 8.7)
15 to 16	─	2.3 (2.0 to 2.6)	4.2 (3.8 to 4.6)	6.2 (5.8 to 6.6)
WHO stage at ART start, n (%)				
1 or 2	1764 (33)	1220 (33)	469 (33)	134 (31)
3 or 4	2503 (47)	1851 (50)	726 (51)	229 (54)
Missing	1097 (20)	655 (17)	239 (17)	65 (15)
Median (IQR) follow‐up from first visit to “transition” (years)	4.8 (3.3 to 6.8)	4.7 (3.1 to 6.7)	6.1 (4.9 to 7.6)	7.5 (6.7 to 8.6)
Median (IQR) follow‐up from first visit to “transition” by age of enrolment in HIV care (years)				
<10	7.7 (6.7 to 9.0)	9.3 (8.5 to 10.6)	11.2 (10.3 to 13.0)	12.9 (12.4 to 14.6)
10 to 14	3.7 (2.8 to 4.7)	5.0 (3.9 to 6.2)	6.7 (5.8 to 7.9)	8.2 (7.6 to 9.1)
15 to 16	─	2.4 (2.2 to 2.7)	4.4 (4.2 to 4.8)	6.5 (6.2 to 6.8)
Number transitioning within same year in facility, n (%)				
≤50	1469 (27)	1233 (32)	610 (42)	279 (63)
51 to 100	969 (18)	607 (16)	337 (23)	161 (37)
101 to 200	683 (12)	509 (13)	283 (19)	─
>200	2395 (43)	1515 (39)	233 (16)	─

ART, antiretroviral therapy; IQR, interquartile range.

a Age used as a proxy for “transition” to adulthood.

**Figure 2:**
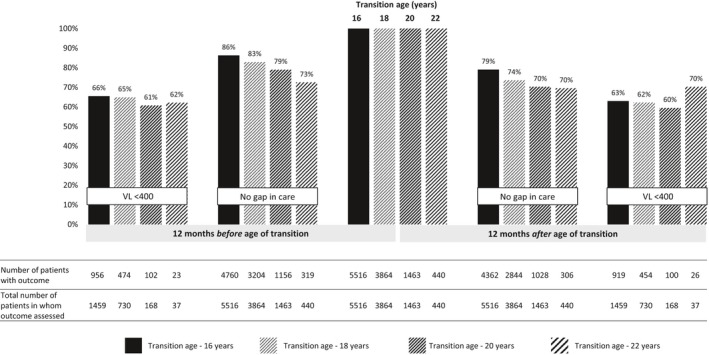
Outcomes across different transition age thresholds.

Eighty‐six percent of AYLH included in the transition‐16y analysis had no gap in care in the 12‐month period pre‐transition, 83% in the transition‐18y, 79% in the transition‐20y and 73% in the transition‐22y age thresholds (Table 2 and Figure 2). In the 12‐month period post‐transition, 79% of AYLH included in the transition‐16y analysis had no gap in care, 74% in the transition‐18y, and 70% in both the transition‐20y and transition‐22y analyses (Table 2 and Figure 2). Compared to the pre‐transition period, the proportion with no gap in care consistently declined post‐transition, across all transition age thresholds (transition‐16y: 86% vs. 79%, difference 7.2 (95% confidence interval (CI) 6.0 to 8.4); transition‐18y: 83% vs. 74%, difference 9.3 (7.8 to 10.9); transition‐20y: 79% vs. 70%, difference 8.7 (6.1 to 11.4); transition‐22y: 73% vs. 70%, difference 3.0 (−2.4 to 8.4)) (Table 2 and Figure 2). Comparing the pre‐ and post‐transition periods, we found no differences in the proportions of patients with viral load measurements (e.g. transition‐18y: 82% vs. 81%, difference 1.1 (−1.8 to 4.1)) or proportions with viral suppression (e.g. transition‐18y: 65% vs. 62%, difference 2.7 (−1.0 to 6.5)). This was observed throughout most transition age thresholds (Table 2 and Figure 2).

**Table 2:** Outcomes across different transition age thresholds

Outcomes	“Transition” at 16 years (N = 5516)	“Transition” at 18 years (N = 3864)	“Transition” at 20 years (N = 1463)	“Transition” at 22 years (N = 440)
No gap in care 12 months *before* age of transition	86%	83%	79%	73%
No gap in care 12 months *after* age of transition	79%	74%	70%	70%
Difference (95% CI)	7.2 (6.0 to 8.4)	9.3 (7.8 to 10.9)	8.7 (6.1 to 11.4)	3.0 (−2.4 to 8.4)

HIV‐RNA viral load done^a^	(N = 1952)	(N = 1040)	(N = 280)	(N = 66)
18 months *before *age of transition	84%	82%	78%	77%
18 months *after* age of transition	84%	81%	74%	68%
Difference (95% CI)	−0.05 (−2.0 to 1.9)	1.1 (−1.8 to 4.1)	3.9 (−3.1 to 10.9)	9.0 (−6.2 to 24.4)

HIV‐RNA <400 copies/mL^b^	(N = 1459)	(N = 730)	(N = 168)	(N = 37)
18 months *before* age of transition	66%	65%	61%	62%
18 months *after* age of transition	63%	62%	60%	70%
Difference (95% CI)	2.5 (0.03 to 5.0)	2.7 (−1.0 to 6.5)	1.2 (−6.8 to 9.2)	−8.1 (−24.6 to 8.4)

CI, confidence interval.

a Limited to patients within facilities with annual routine viral load monitoring

b limited to patients with viral load measurements done before and after the respective age threshold.

**Figure 3:**
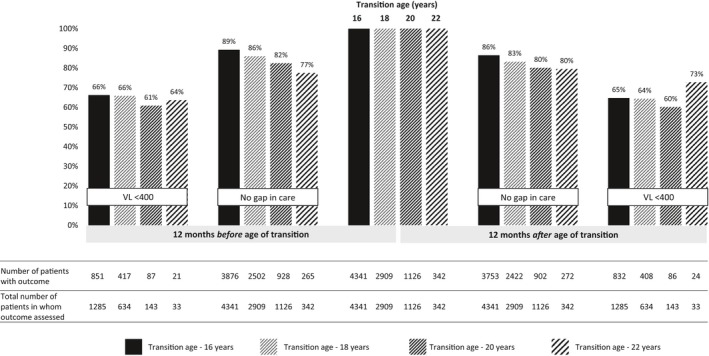
Outcomes across different transition age thresholds – restricted to patients still in care at the end of follow‐up.

In the sensitivity analyses comparing the pre‐ and post‐transition periods among patients still in care at the time of database closure, we found that the proportion with no gap in care also consistently declined post‐ when compare to pre‐transition at the transition‐16y and transition‐18y thresholds (transition‐16y: 89% vs. 86%, difference 2.8 (1.6 to 4.0); transition‐18y: 86% vs. 83%, difference 2.7 (1.2 to 4.3)), but there were no differences at the transition‐20y and transition‐22y thresholds (transition‐20y: 82% vs. 80%, difference 2.3 (−0.5 to 5.1); transition‐22y: 77% vs. 80%, difference −3.0 (−7.7 to 3.6)) (Table 3 and Figure 3). Across all transition‐age thresholds, there were no differences in the proportions of patients with viral load measurements or proportions virally suppressed between the pre‐ and post‐transition periods (Table 3 and Figure 3). In further analyses assuming transition at age 15 years, we found very similar results to those from the transition‐16y analysis (Table S1).

**Table 3:** Outcomes across different transition age thresholds to restricted to patients still in care at the end of follow‐up

Outcomes	“Transition” at 16 years (N = 4341)	“Transition” at 18 years (N = 2909)	“Transition” at 20 years (N = 1126)	“Transition” at 22 years (N = 342)
No gap in care 12 months *before* age of transition	89%	86%	82%	77%
No gap in care 12 months *after* age of transition	86%	83%	80%	80%
Difference (95% CI)	2.8 (1.6 to 4.0)	2.7 (1.2 to 4.3)	2.3 (−0.5 to 5.1)	−3.0 (−7.7 to 3.6)

HIV‐RNA viral load done^a^	(N = 1706)	(N = 888)	(N = 235)	(N = 56)
18 months *before* age of transition	84%	83%	78%	77%
18 months *after* age of transition	85%	82%	76%	71%
Difference (95% CI)	−0.5 (−2.6 to 1.6)	0.9 (−2.3 to 4.1)	2.1 (−5.6 to 9.9)	5.4 (−10.8 to 21.5)

HIV‐RNA <400 copies/mL^b^	(N = 1285)	(N = 634)	(N = 143)	(N = 33)
18 months *before* age of transition	66%	66%	61%	64%
18 months *after* age of transition	65%	64%	60%	73%
Difference (95% CI)	1.5 (−1.2 to 4.1)	1.4 (−2.6 to 5.5)	0.7 (−8.1 to 9.5)	−9.1 (−27.5 to 9.3)

CI, confidence interval.

a Limited to patients still in care at the end of follow‐up within facilities with annual routine viral load monitoring;

b limited to patients still in care at the end of follow‐up with viral load measurements done before and after the respective age threshold.

**Figure 4:**
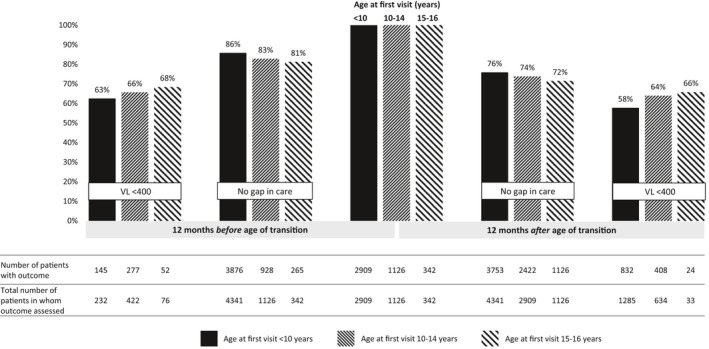
Outcomes in the 12 months *before* and 12 months *after* transition age 18 years, by age of enrolment into HIV care.

We found no difference in the proportion of patients virally suppressed between the pre‐ and post‐transition periods among AYLH included in this analysis with assumed perinatal HIV (transition‐18y: 63% vs. 58%, difference 4.7 (−2.3 to 11.7)) and those with non‐perinatal HIV (transition‐18y: 66% vs. 64%, difference 1.7 (−3.4 to 6.7) and 68% vs. 66%, difference 2.6 (−9.0 to 14.2)) (Figure 4 and Table S2). However, as with the whole cohort, proportion with no gap in care declined post‐transition when compared to pre‐transition in both groups (Figure 4 and Table S2). Results for the transition‐16y and transition‐20y age thresholds are shown in Table S2 and Figure S1.

Using log‐binomial regression, patients were consistently more likely to be retained post‐transition if they had no gap in care in the preceding year, across all transition‐age thresholds (transition‐16y: adjusted risk ratio (aRR) 1.72 (95% CI 1.60 to 1.86); transition‐18y: aRR 1.76 (1.61 to 1.92); transition‐20y: aRR 1.75 (1.53 to 2.01); transition‐22y: aRR 1.47 (1.21 to 1.78) (Table 4). Sex was not associated with post‐transition retention throughout all transition‐age thresholds. Also, for certain transition‐age thresholds, the number of youth transitioning at the same time within a clinic was associated with retention. Compared to patients transitioning with ≤50 other patients within the same year in the clinic, those transitioning with 51 to 100 other patients were slightly more likely to be retained at the transition‐16y (aRR 1.06 (1.03 to 1.09)) and transition‐18y (aRR 1.04 (1.01 to 1.08)) age thresholds; those transitioning with 101 to 200 other patients were less likely to be retained at the transition‐18y (aRR 0.89 (0.84 to 0.94)) and transition‐20y (aRR 0.80 (0.72 to 0.88)) age thresholds, and those transitioning with >200 other patients were less likely to be retained across the transition‐16y: aRR 0.88 (0.85 to 0.91), transition‐18y: aRR 0.79 (0.76 to 0.83), and transition‐20y: aRR 0.74 (0.66 to 0.84) age thresholds (Table 4). Similar results were also observed in the analyses restricted to AYLH still in care at the end of follow‐up (Table 5).

**Table 4:** Regression of predictors of retention in the 12 months *after* the age of transition across transition‐age thresholds: 16, 18, 20 and 22 years

Characteristic	Transition at 16 years (N = 5516) aRR (95% CI)	Transition at 18 years (N = 3864) aRR (95% CI)	Transition at 20 years (N = 1463) aRR (95% CI)	Transition at 22 years (N = 440) aRR (95% CI)
Female (vs. male)	0.99 (0.97 to 1.01)	0.99 (0.96 to 1.02)	1.01 (0.96 to 1.07)	0.99 (0.89 to 1.11)
Age at enrolment into HIV care (years)				
<10	ref	ref	ref	ref
10 to 14	1.00 (0.98 to 1.03)	1.02 (0.98 to 1.07)	1.15 (0.98 to 1.35)	1.50 (0.76 to 2.94)
15 to 16	–	1.02 (0.97 to 1.08)	1.18 (1.00 to 1.39)	1.55 (0.79 to 3.04)
Number transitioning within same year in facility, n (%)				
≤50	ref	ref	ref	ref
51 to 100	1.06 (1.03 to 1.09)	1.04 (1.01 to 1.08)	0.95 (0.89 to 1.01)	0.85 (0.74 to 0.98)
101 to 200	1.04 (1.01 to 1.07)	0.89 (0.84 to 0.94)	0.80 (0.72 to 0.88)	–
>200	0.88 (0.85 to 0.91)	0.79 (0.76 to 0.83)	0.74 (0.66 to 0.84)	–
≥2 visits in 12 months *before* age of transition	1.72 (1.60 to 1.86)	1.76 (1.61 to 1.92)	1.75 (1.53 to 2.01)	1.47 (1.21 to 1.78)

aRR, adjusted risk ratio; CI, confidence interval.

**Table 5:** Regression of predictors of retention in the 12 months *after* the age of transition across transition‐age thresholds: 16, 18, 20 and 22 years – restricted to patients still in care at the end of follow‐up

Characteristic	Transition at 16 years (N = 4341) aRR (95% CI)	Transition at 18 years (N = 2909) aRR (95% CI)	Transition at 20 years (N = 1126) aRR (95% CI)	Transition at 22 years (N = 342) aRR (95% CI)
Female (vs. male)	0.99 (0.97 to 1.01)	1.01 (0.98 to 1.03)	1.00 (0.96 to 1.05)	1.06 (0.96 to 1.18)
Age at enrolment into HIV care (years)				
<10	ref	ref	ref	ref
10 to 14	1.00 (0.98 to 1.02)	1.01 (0.97 to 1.04)	1.04 (0.92 to 1.16)	1.10 (0.71 to 1.72)
15 to 16	–	1.00 (0.96 to 1.04)	1.05 (0.93 to 1.18)	1.13 (0.73 to 1.77)
Number transitioning within same year in facility, n (%)				
≤50	ref	ref	ref	ref
51 to 100	1.06 (1.04 to 1.08)	1.02 (0.99 to 1.05)	0.91 (0.85 to 0.97)	0.87 (0.74 to 1.02)
101 to 200	0.93 (0.89 to 0.97)	0.85 (0.81 to 0.89)	0.81 (0.75 to 0.88)	–
>200	0.93 (0.90 to 0.95)	0.88 (0.85 to 0.92)	–	–
≥2 visits in 12 months *before* age of transition	1.44 (1.33 to 1.55)	1.57 (1.43 to 1.72)	1.47 (1.29 to 1.67)	1.32 (1.10 to 1.60)

aRR, adjusted risk ratio; CI, confidence interval.

## 4 Discussion

To the best of our knowledge, this is the first analysis to examine gaps in care and viral suppression outcomes in the pre‐and post‐transition periods for adolescents and young adults living with HIV transitioning to adulthood without transferring care within facilities in Southern Africa. We demonstrate that across multiple transition‐age thresholds, retention of youth in care declined after transition, with this trend worsening as the age of transition increased from 16 to 22 years. Our data support the conclusion that adolescents and young adults are at an increased risk of disengaging from care as they reach the ages when they are expected to take responsibility for their own care and are managed as “adults.”

Our findings show a decline in the proportion of youth who consistently remain in care after reaching transition ages, with 79% having no gaps in care in the 12 months after transition‐16y and 70% after transition‐22y. While our study used age as a proxy of transition to adulthood, our findings are consistent with studies from other cohorts that have shown sub‐optimal engagement in care as adolescents enter adulthood 7, 8, 11, 20, 21. For example, one study in the Netherlands reported an increase in the mean number of individual yearly missed appointments as adolescents aged up to young adulthood 22, while another in Zimbabwe showed that loss to follow‐up among those who started ART as older adolescents (15 to 19 years) nearly doubled as they aged up to the ages of 20 to 24 years 23. Another analysis that looked at loss to follow‐up rates within the US HIV Research Network (HIVRN) cohort reported that 11% of patients were lost to follow‐up in the year after their 18^th^ birthday and 20% were lost after their 22^nd^ birthday 24, 25. Other studies from settings were adolescents and young adults transfer to adult care as part of the transition process have reported worse outcomes 11, 12. However, direct comparison with our results is difficult given that patients in our study remained at the same facility over time.

While post‐transition virologic suppression rates were low in our cohorts (60% to 70%), they were similar to the pre‐transition rates. For adolescents who have transferred to adult clinics, virologic suppression rates in adult clinics have not differed substantially from those in the paediatric setting 16. Notably, poor treatment adherence in paediatric care has been reported to be a reliable predictor of adherence in adult care 22. This emphasizes the importance of taking available opportunities to proactively address adherence issues early in care in order to improve later adherence and ensure adolescents have effective tools they can take into adult life.

While the assumed mode of HIV acquisition and sex were not associated with post‐transition retention, the strongest predictor of post‐transition retention was prior poor engagement in care. Youth with gaps in care have been shown to be at risk of being lost to follow‐up as they grow older 25. Furthermore, we found that for certain transition‐age thresholds, the number of patients transitioning within the same year in the clinic impacted retention. Compared to youth transitioning with ≤50 others, youth transitioning with 51 to 100 others did better at the transition‐16y and transition‐18y thresholds, those transitioning with 101 to 200 others did worse at the transition‐18y and transition‐20y thresholds, and those transitioning with >200 other patients did worse across all transition‐age thresholds. Although these results are difficult to interpret, it is possible that clinics with few transitioning patients are not adequately equipped to deal with the transition process. In contrast, clinics with too many patients may lack enough staff and time to support individualized and adolescent‐friendly care for youth. An earlier survey of facilities providing care to adolescents living with HIV in sub‐Saharan Africa showed that half of facilities had no guidelines or protocols for managing adolescent transition 14.

The major caveat to the interpretation of this study is that we used age as a proxy for transition to autonomy in the context of clinical care where youth remained in the same care facility as they aged and without knowing when they actually became responsible for their own individual health management. Also, we did not include a comparison with those who were known to have been transferred out of care for other reasons, who were silently transferred (e.g. remained in care at another facility through an undocumented transfer) or who were LTFU before each respective age thresholds. Our definition of “retention” was pragmatic but relatively crude and we may have misclassified patients who had temporary gaps in care but later re‐engaged with HIV care services. In addition, our analysis was limited to variables that were available in routinely collected data and we could not measure specific factors related to independence or self‐management of care such as the ability to make appointments, attending clinic appointments without a parent or caregiver, and transition‐related processes which may entail changes in the clinic days or times, or movements to a different section of the facility, without transfer of care. Despite these limitations, given that our analysis used routinely collected longitudinal data, our findings may be more generalizable to settings with similar transition processes. By studying engagement in care and viral suppression in the year before and after transition, we were able to more fully characterize care experiences of youth as a continuum, showing how care‐related behaviours earlier in life impact those later in life.

For adolescents and young adults living with HIV, the period of healthcare transition is a particularly vulnerable one that is associated with greater risk of disengagement from care. We found that gaps in care earlier in adolescence and young adulthood are a marker for worse outcomes later. There is an urgent need for timely interventions for AYLH with gaps in care before they reach transition ages and for models of care tailored to the needs of transitioning adolescents and young adults. For AYLH managed within facilities where transition to adulthood does not entail a physical transfer of care to an adult HIV clinic, greater awareness of the risk of poorer outcomes and investment of human and technical resources are needed to ensure they successfully adapt to changing expectations for their care.

## 5 Conclusions

Our analysis demonstrates that AYLH with gaps in care need targeted support to prevent non‐retention as they age and take on greater responsibility for their healthcare. We noted with concern the low virologic suppression rates both in the pre‐ and post‐transition periods.

## Competing interests

Authors have no competing interests to declare.

## Authors' contributions

MD, AS, PT, AJ and IC conceptualized the concept. PT conducted the analysis and drafted the manuscript. SP, KS, SS, IC, GF, MAH, KT, HR, JB, MF are members of the IeDEA‐SA Steering Group and commented and provided feedback on the manuscript. All authors have read and approved the final manuscript.

## Supporting information


**Table S1.** Outcomes at transition age threshold 15 years
**Table S2.** Outcomes at different transition age thresholds by age of enrolment into HIV care
**Figure S1.** Outcomes at different transition age thresholds by age of enrolment into HIV care: at transition age thresholds (a) 16 years (b) 20 years.

## References

[1] UNAIDS . 2018 Estimates. Geneva, Switzerland: UNAIDS; 2018 [cited 2019 Jun 15]. Available from: http://aidsinfo.unaids.org/

[2] Nachega JB , Hislop M , Nguyen H , Dowdy DW , Chaisson RE , Regensberg L , et al. Antiretroviral therapy adherence, virologic and immunologic outcomes in adolescents compared with adults in southern Africa. J Acquir Immune Defic Syndr. 2009;51(1):65–71. 19282780 10.1097/QAI.0b013e318199072e PMC2674125

[3] Evans D , Menezes C , Mahomed K , Macdonald P , Untiedt S , Levin L , et al. Treatment outcomes of HIV‐infected adolescents attending public‐sector HIV clinics across Gauteng and Mpumalanga, South Africa. AIDS Res Hum Retroviruses. 2013;29(6):892–900. 23373540 10.1089/aid.2012.0215 PMC3653371

[4] Lamb MR , Fayorsey R , Nuwagaba‐Biribonwoha H , Viola V , Mutabazi V , Alwar T , et al. High attrition before and after ART initiation among youth (15–24 years of age) enrolled in HIV care. AIDS. 2014;28(4):559. 24076661 10.1097/QAD.0000000000000054 PMC4517438

[5] Weigel R , Estill J , Egger M , Harries A , Makombe S , Tweya H , et al. Mortality and loss to follow‐up in the first year of ART: Malawi national ART programme. AIDS. 2012;26(3):365–73. 22095194 10.1097/QAD.0b013e32834ed814 PMC3811026

[6] Marcus RBJ , Kranzer K . Loss to follow‐up in children and adolescents with increasing age in South Africa. 9th International Workshop on HIV Pediatrics. Paris, France: 2017.

[7] Ryscavage P , Macharia T , Patel D , Palmeiro R , Tepper V . Linkage to and retention in care following healthcare transition from pediatric to adult HIV care. AIDS Care. 2016;28(5):561–5. 26766017 10.1080/09540121.2015.1131967

[8] Fish R , Judd A , Jungmann E , O'Leary C , Foster C . Mortality in perinatally HIV‐infected young people in England following transition to adult care: an HIV Young Persons Network (HYPNet) audit. HIV Med. 2014;15(4):239–44. 24112550 10.1111/hiv.12091

[9] Izzo I , Quiros‐Roldan E , Saccani B , Chiari E , Casari S , Foca E , et al. Perinatally HIV‐infected youths after transition from pediatric to adult care, a single‐center experience from Northern Italy. AIDS Res Hum Retroviruses. 2018;34(3):241–3. 29061072 10.1089/AID.2017.0120

[10] Hansudewechakul R , Pongprapass S , Kongphonoi A , Denjanta S , Watanaporn S , Sohn AH . Transition of Thai HIV‐infected adolescents to adult HIV care. J Int AIDS Soc. 2015;18:20651. 26637523 10.7448/IAS.18.1.20651 PMC4670453

[11] Hope R , Judd A , Foster C , Prime K , Jungmann E , Tookey P , et al. editors. Clinical outcomes in adults with perinatal HIV after transfer from pediatric care. conference on retroviruses and opportunistic infections. Boston, USA; 2016.

[12] Abreu‐Perez RL‐NL , Beck‐Sague C . Where are they now? Mortality, loss to follow‐up and viral suppression in perinatally HIVinfected (PHIV) posttransition young adult antiretroviral therapy (ART) patients in the Dominican Republic (DR): 2004–2015. 21st International AIDS Conference. Durban: South Africa;2016.

[13] Dahourou DL , Gautier‐Lafaye C , Teasdale CA , Renner L , Yotebieng M , Desmonde S , et al. Transition from paediatric to adult care of adolescents living with HIV in sub‐Saharan Africa: challenges, youth‐friendly models, and outcomes. J Int AIDS Soc. 2017;20 Suppl 3:21528. 28530039 10.7448/IAS.20.4.21528 PMC5577723

[14] Mark D , Armstrong A , Andrade C , Penazzato M , Hatane L , Taing L , et al. HIV treatment and care services for adolescents: a situational analysis of 218 facilities in 23 sub‐Saharan African countries. J Int AIDS Soc. 2017;20 Suppl 3:21591. 28530038 10.7448/IAS.20.4.21591 PMC5719719

[15] Judd A , Collins IJ , Parrott F , Hill T , Jose S , Ford D , et al. Growing up with perinatal HIV: changes in clinical outcomes before and after transfer to adult care in the UK. J Int AIDS Soc. 2017;20 Suppl 3:21577. 28530042 10.7448/IAS.20.4.21577 PMC5577702

[16] Hussen SA , Chakraborty R , Knezevic A , Camacho‐Gonzalez A , Huang E , Stephenson R , et al. Transitioning young adults from paediatric to adult care and the HIV care continuum in Atlanta, Georgia, USA: a retrospective cohort study. J Int AIDS Soc. 2017;20(1):21848. 28872281 10.7448/IAS.20.1.21848 PMC5705166

[17] Davies MSS , Phiri S , Rabie H , Eley B , Fatti G . What does adolescent transition mean in sub‐Saharan Africa? Predictors of transfer in Southern African perinatally HIV‐infected adolescents: oral abstracts of the 21st International AIDS Conference 18–22 July 2016, Durban, South Africa. J Int AIDS Soc. 2016;19 6 Suppl 5:21264.27460772

[18] Egger M , Ekouevi DK , Williams C , Lyamuya RE , Mukumbi H , Braitstein P , et al. Cohort Profile: the international epidemiological databases to evaluate AIDS (IeDEA) in sub‐Saharan Africa. Int J Epidemiol. 2012;41(5):1256–64. 21593078 10.1093/ije/dyr080 PMC3465765

[19] Collaborative Initiative for Paediatric HIVE, Research Global Cohort C , Slogrove AL , Schomaker M , Davies M‐A , Williams P , Balkan S , et al. The epidemiology of adolescents living with perinatally acquired HIV: a cross‐region global cohort analysis. PLoS Medicine. 2018;15:e1002514. 29494593 10.1371/journal.pmed.1002514 PMC5832192

[20] Kakkar F , Van der Linden D , Valois S , Maurice F , Onnorouille M , Lapointe N , et al. Health outcomes and the transition experience of HIV‐infected adolescents after transfer to adult care in Quebec, Canada. BMC Pediatr. 2016;16:109. 27457719 10.1186/s12887-016-0644-4 PMC4960665

[21] Tepper V , Zaner S , Ryscavage P . HIV healthcare transition outcomes among youth in North America and Europe: a review. J Int AIDS Soc. 2017;20 Suppl 3:21490. 28530041 10.7448/IAS.20.4.21490 PMC5577703

[22] Weijsenfeld AM , Smit C , Cohen S , Wit FWNM , Mutschelknauss M , van der Knaap LC , et al. Virological and social outcomes of HIV‐infected adolescents and young adults in the Netherlands before and after transition to adult care. Clin Infect Dis. 2016;63(8):1105–12. 27439528 10.1093/cid/ciw487

[23] Kranzer K , Bradley J , Musaazi J , Nyathi M , Gunguwo H , Ndebele W , et al. Loss to follow‐up among children and adolescents growing up with HIV infection: age really matters. J Int AIDS Soc. 2017;20(1):21737. 28715158 10.7448/IAS.20.1.21737 PMC5577636

[24] Agwu A , Althoff K , Rutstein R , Korthuis P , Berry S , Gaur A , et al. editors. Factors associated with falling out of care for older adolescents in the HIV Research Network. 19th International AIDS Conference. Washington, DC; 2012.

[25] Agwu A , Lee L , Fleishman JA , Voss C , Yehia B , Althoff K , et al. Aging and loss to follow‐up among youth living with human immunodeficiency virus in the HIV Research Network. J Adolesc Health. 2015;56(3):345–51. 25703322 10.1016/j.jadohealth.2014.11.009 PMC4378241

