# Supplementary material for: Characterizing the double‐sided cascade of care for adolescents living with HIV transitioning to adulthood across Southern Africa
Source: J Int AIDS Soc. 2020 Jan 30;23(1):e25447. doi: 10.1002/jia2.25447 (PMC6992508; doi:10.1002/jia2.25447)
Supplement: Supplementary file 1 — Table S1. Outcomes at transition age threshold 15 years Table S2. Outcomes at different transition age thresholds by age of enrolment into HIV care Figure S1. Outcomes at different transition age thresholds by age of enrolment into HIV care: at transition age thresholds (a) 16 years (b) 20 years. [file JIA2-23-e25447-s001.docx]

**Supplementary Table 1:** Outcomes at transition age threshold 15 years

| **Outcomes** | **“Transition” at 15 years**  **(N=7836)** | **“Transition” at 15 years – restricted to patients still in care at end of follow-up (N=5028)** |
| --- | --- | --- |
| No gap in care 12 months *before* age of transition | 87% | 88% |
| No gap in care 12 months *after* age of transition | 80% | 87% |
| Difference (95% CI) | 7.0 (6.0 – 8.0) | 1.5 (0.4 – 2.6) |
| **HIV-RNA viral load done**^†^ | **(N=2872)** | **(N=1878)** |
| 18 months before age of transition | 83% | 80% |
| 18 months after age of transition | 83% | 82% |
| Difference (95% CI) | -0.08 (-2.5 – 0.9) | -2.2 (-4.5 – 0.1) |
| **HIV-RNA <400 copies/mL**^§^ | **(N=2076)** | **(N=1288)** |
| 18 months before age of transition | 70% | 67% |
| 18 months after age of transition | 63% | 61% |
| Difference (95% CI) | 6.3 (4.3 – 8.4) | 6.0 (3.3 – 8.7) |

CI: confidence interval

^‡^ limited to patients within facilities with annual routine viral load monitoring

^§^ limited to patients with viral load measurements done before and after the respective age threshold

**Supplementary Figure 1.** Outcomes at different transition age thresholds by age of enrolment into HIV care: at transition age thresholds a) 16 years b) 20 years.


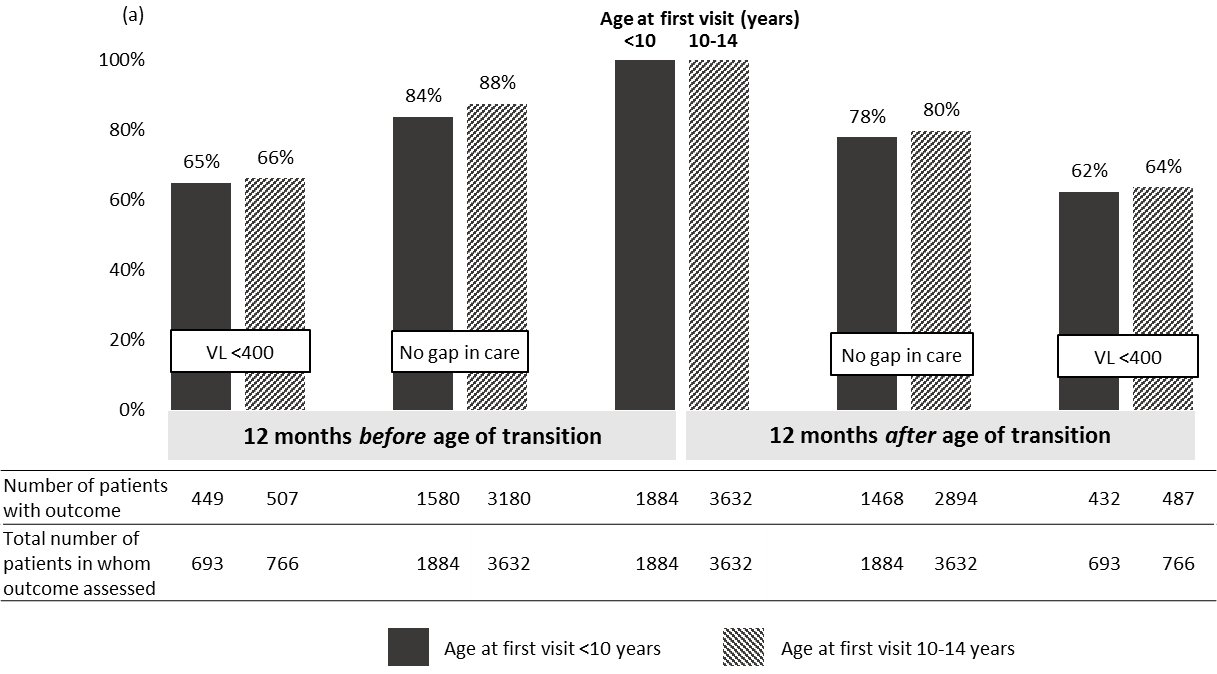


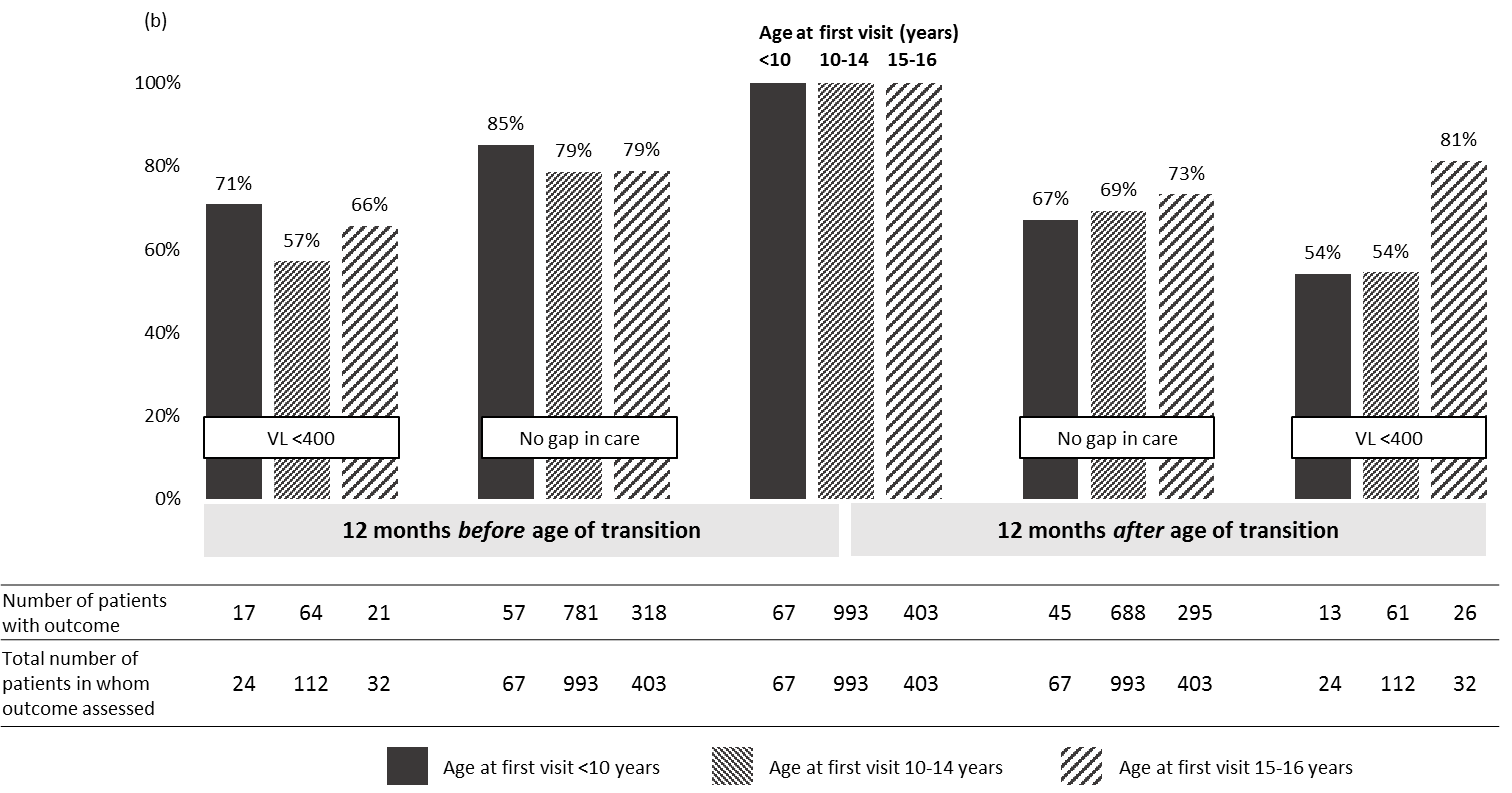


| **Supplementary Table 2.** Outcomes at different transition age thresholds by age of enrolment into HIV care | | | | | | | | | | |
| --- | --- | --- | --- | --- | --- | --- | --- | --- | --- | --- |
| **Outcomes** | *Transition at 16 years (N=5516)* | |  | *Transition at 18 years (N=3864)* | | |  | *Transition at 20 years (N=1463)* | | |
|  | **Age at enrolment into HIV care** | |  | **Age at enrolment into HIV care** | | |  | **Age at enrolment into HIV care** | | |
|  | **<10 years**  **(n=1884)** | **10-14 years**  **(n=3632)** |  | **<10 years**  **(n=543)** | **10-14 years**  **(n=2489)** | **15-16 years**  **(n=832)** |  | **<10 years**  **(n=67)** | **10-14 years**  **(n=993)** | **15-16 years**  **(n=403)** |
| No gap in care 12 months *before* age of transition | 84% | 88% |  | 86% | 83% | 81% |  | 85% | 79% | 79% |
| No gap in care 12 months after *age* of transition | 78% | 80% |  | 76% | 74% | 72% |  | 67% | 69% | 73% |
| Difference (95% CI) | 5.9 (3.9-8.0) | 7.9 (6.4-9.3) |  | 9.9 (6.1-13.8) | 9.0 (7.1-11.0) | 9.7 (6.3-13.2) |  | 17.9 (5.5-30.3) | 9.4 (6.0-12.7) | 5.7 (0.8-10.6) |
| **HIV viral load done**^†^ | **(n=886)** | **(n=1066)** |  | **(n=284)** | **(n=608)** | **(n=148)** |  | **(n=37)** | **(n=177)** | **(n=66)** |
| 18 months *before* age of transition | 87% | 82% |  | 91% | 81% | 66% |  | 92% | 81% | 62% |
| 18 months *after* age of transition | 87% | 82% |  | 88% | 80% | 70% |  | 68% | 77% | 71% |
| Difference (95% CI) | -0.6 (-3.4-2.3) | 0.4 (-2.5-3.2) |  | 2.8 (-2.1-7.7) | 1.6 (-2.3-5.6) | -4.1 (-13.9-5.8) |  | 24.3 (5.9-42.8) | 4.5 (-4.3-13.3) | -9.1 (-25.0-6.8) |
| **HIV-RNA<400 copies/mL**^§^ | **(n=693)** | **(n=766)** |  | **(n=232)** | **(n=422)** | **(n=76)** |  | **(n=24)** | **(n=112)** | **(n=32)** |
| 18 months *before* age of transition | 65% | 66% |  | 63% | 66% | 68% |  | 71% | 57% | 66% |
| 18 months *after* age of transition | 62% | 64% |  | 58% | 64% | 66% |  | 54% | 54% | 81% |
| Difference (95% CI) | 2.4 (-1.2-6.1) | 2.6 (-0.9-6.1) |  | 4.7 (-2.3-11.7) | 1.7 (-3.4-6.7) | 2.6 (-9.0-14.2) |  | 16.7 (-6.4-39.7) | 2.7 (-7.3-12.7) | -15.6 (-34.0-2.8) |
| ^†^ limited to patients within facilities with annual routine viral load monitoring  ^§^ limited to patients with viral load measurements done before and after the respective age threshold | | | | | | | | | | |
